# Crystal structure of (4*Z*)-4-[(2*E*)-3-(4-chloro­phen­yl)-1-hy­droxy­prop-2-en-1-yl­idene]-5-methyl-2-phenyl-1*H*-pyrazol-5(4*H*)-one

**DOI:** 10.1107/S205698901500883X

**Published:** 2015-05-13

**Authors:** Muhammad Shahid, Munawar Ali Munawar, Muhammad Nawaz Tahir, Muhammad Salim, Khizar Iqbal Malik

**Affiliations:** a Department of Chemistry, University of the Punjab, Lahore, Punjab, Pakistan; b Department of Physics, University of Sargodha, Sargodha, Punjab, Pakistan

**Keywords:** crystal structure, intra­molecular O—H⋯O hydrogen bond, C—H⋯O inter­actions, π–π stacking inter­actions

## Abstract

In the the asymmetric unit of the title compound, C_19_H_15_ClN_2_O_2_, there are two symmetry-independent mol­ecules, which adopt similar conformations. The largest difference is observed in the dihedral angles between the phenyl and the pyrazole fragments [17.00 (12) and 23.42 (10)°]. A strong intra­molecular O—H⋯O hydrogen bond with the *S* (6) motif is observed in both mol­ecules. Pairs of π–π stacking inter­actions between the phenyl groups [centroid–centroid distances = 3.6627 (13) and 3.7156 (14) Å] assemble the mol­ecules into two types of centrosymmetric dimers. Weak C—H⋯O inter­actions connect mol­ecules into chains along the *b* axis.

## Related literature   

For related structures and background, see: Chaudhry *et al.* (2012 ▸); Holzer *et al.* (1999 ▸); Malik *et al.* (2009 ▸).
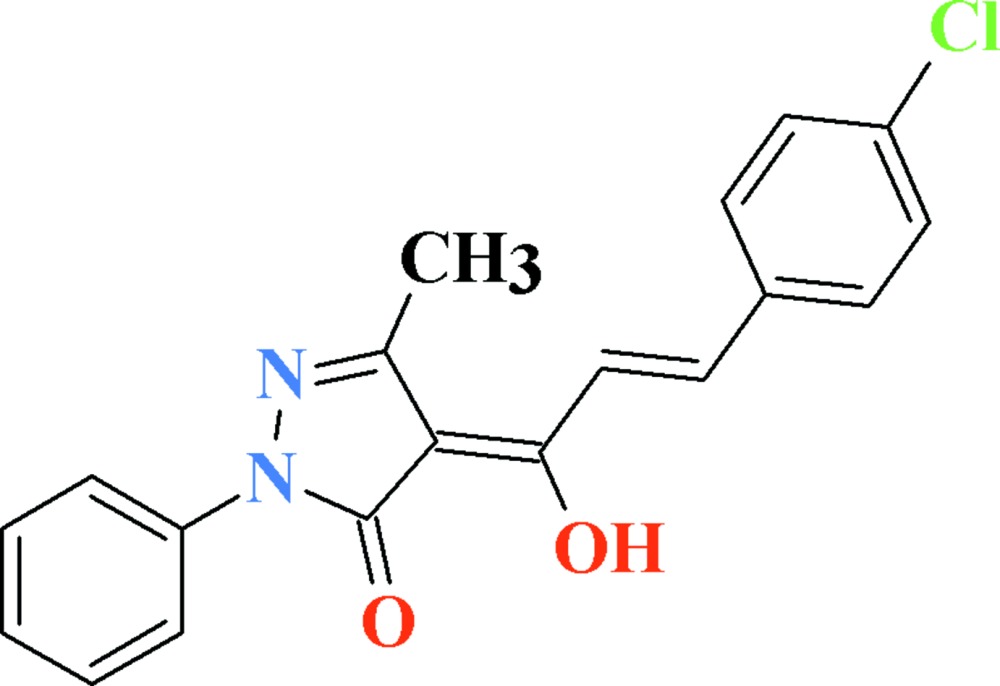


## Experimental   

### Crystal data  

C_19_H_15_ClN_2_O_2_
*M* *_r_* = 338.78
Triclinic, 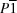

*a* = 11.3207 (6) Å
*b* = 11.4044 (6) Å
*c* = 15.2839 (9) Å
α = 70.567 (3)°
β = 70.925 (3)°
γ = 62.621 (2)°
*V* = 1616.40 (16) Å^3^
*Z* = 4
Mo *K*α radiation
μ = 0.25 mm^−1^
*T* = 296 K
0.35 × 0.28 × 0.16 mm

### Data collection  

Bruker Kappa APEXII CCD diffractometer
Absorption correction: multi-scan (*SADABS*; Bruker, 2005 ▸) *T*_min_ = 0.919, *T*_max_ = 0.963
24363 measured reflections
6925 independent reflections
4587 reflections with *I* > 2σ(*I*)
*R*_int_ = 0.038

### Refinement  

*R*[*F* ^2^ > 2σ(*F* ^2^)] = 0.050
*wR*(*F* ^2^) = 0.155
*S* = 1.02
6925 reflections
437 parameters
H-atom parameters constrained
Δρ_max_ = 0.36 e Å^−3^
Δρ_min_ = −0.32 e Å^−3^

### 

Data collection: *APEX2* (Bruker, 2007 ▸); cell refinement: *SAINT* (Bruker, 2007 ▸); data reduction: *SAINT*; program(s) used to solve structure: *SHELXS97* (Sheldrick, 2008 ▸); program(s) used to refine structure: *SHELXL2014* (Sheldrick, 2015 ▸); molecular graphics: *ORTEP-3 for Windows* (Farrugia, 2012 ▸) and *PLATON* (Spek, 2009 ▸); software used to prepare material for publication: *WinGX* (Farrugia, 2012 ▸) and *PLATON*.

## Supplementary Material

Crystal structure: contains datablock(s) global, I. DOI: 10.1107/S205698901500883X/gk2632sup1.cif

Structure factors: contains datablock(s) I. DOI: 10.1107/S205698901500883X/gk2632Isup2.hkl

**Fig. 1.**
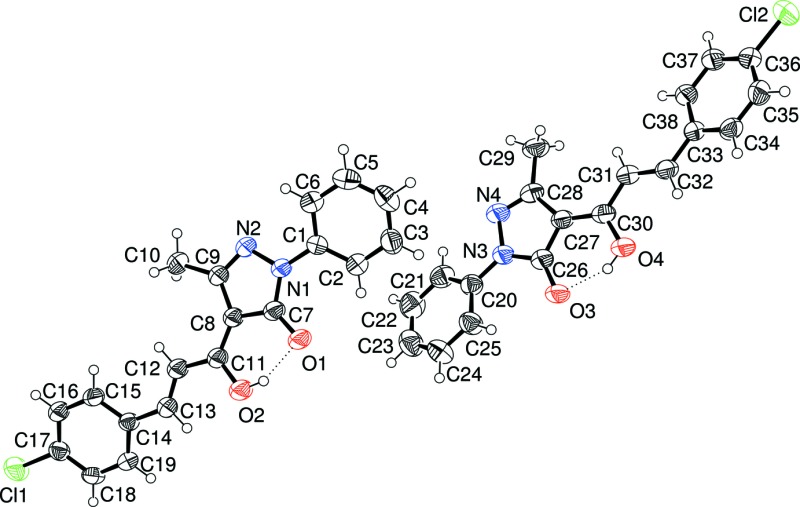
View of the asymmetric unit. The displacement ellipsoids are drawn at the 50% probability level. H-atoms are shown by small circles of arbitrary radii.

. DOI: 10.1107/S205698901500883X/gk2632fig1.tif
View of the asymmetric unit. The displacement ellipsoids are drawn at the 50% probability level. H-atoms are shown by small circles of arbitrary radii.

**Fig. 2.**
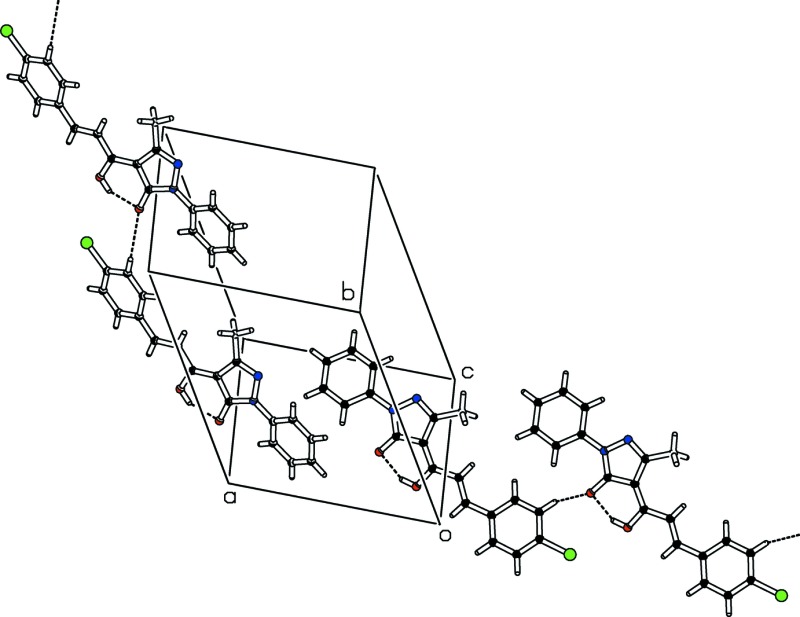
The partial packing (PLATON; Spek, 2009) showing fragments of two chains via C—H···O interactions.

PLATON . DOI: 10.1107/S205698901500883X/gk2632fig2.tif
The partial packing (*PLATON*; Spek, 2009) showing fragments of two chains via C—H⋯O inter­actions.

CCDC reference: 1063448

Additional supporting information:  crystallographic information; 3D view; checkCIF report

## Figures and Tables

**Table 1:** Hydrogen-bond geometry (, )

*D*H*A*	*D*H	H*A*	*D* *A*	*D*H*A*
O2H2*A*O1	0.82	1.78	2.542(2)	153
C16H16O1^i^	0.93	2.47	3.239(2)	140
O4H4*A*O3	0.82	1.78	2.540(2)	153
C37H37O3^ii^	0.93	2.61	3.314(3)	133

Symmetry codes: (i) 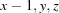; (ii) 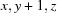.

## supplementary crystallographic information

### S1. Comment

The crystal structures of 5-methyl-2-phenyl-4-((*E*)-3-phenyl-2-hydroxy- prop-2-enylidene)-1,2-dihydro-3*H*-pyrazol-3-one (Holzer *et al.*, 1999), (4*Z*)-4-((2*E*)-1-hydroxy-3-(4-methoxyphenyl)prop-2-en-1- ylidene)-3-methyl-1-phenyl-1*H*-pyrazol-5(4*H*)-one (Malik *et al.*, 2009) and (4*Z*)-4-((2*E*)-1-hydroxy-3-(3-nitrophenyl)prop- 2-en-1-ylidene)-3-methyl-1-(4-methylphenyl)-1*H*-pyrazol-5(4*H*)-one (Chaudhry *et al.*, 2012) have been published which are related to the title compound (I, Fig. 1). The title compound was synthesized for the biological studies as well as for the preparation of different metal complexes.

There are two symmetry independent molecules in the asymmetric unit. In one molecule, the benzene ring *A* (C1—C6), the 5-methyl-2,4-dihydro -3*H*-pyrazol-3-one moiety *B* (C7–C10/N1/N2/O1), prop-2-en-1-ol group *C* (C11/C12/C13/O2) and chlorobenzene group *D* (C14—C19/CL1) are planar with r. m. s. deviations of 0.0062, 0.0131, 0.0319 and 0.0050 Å, respectively. The dihedral angle between A/B, B/C, C/D and A/D is 23.21 (9), 6.20 (10), 18.48 (12) and 1.15 (12)°, respectively. In the second molecule, similar groups *i.e.* the benzene ring *E* (C20—C25), the 5-methyl-2,4- dihydro-3*H*-pyrazol-3-one moiety *F* (C26–C29/N3/N4/O3), prop-2-en-1-ol group *G* (C30/C31/C32/O4) and chlorobenzene group *H* (C33—C38/CL2) are planar with r. m. s. deviations of 0.0041, 0.0080, 0.0272 and 0.0144 Å, respectively. The dihedral angle between E/F, F/G, G/H and E/H is 16.74 (10), 5.66 (12), 13.81 (13) and 3.07 (13)°, respectively. There exist strong intramolecular hydrogen bond O—H····O (Table 1, Fig. 1) forming *S*(6) ring motif (Bernstein *et al.*, 1995) in each molecule. The molecules are interlinked with each other due to C—H···O interactions (Table 1, Fig. 2). There exist π–π interactions with a distance of 3.6627 (13) Å between the centeroids of *Cg*2—*Cg*3^i^ and *Cg*3—*Cg*2^i^ [i = -*x*, -*y*, 1 - *z*], where *Cg*2 and *Cg*3 are the centroids of the benzene ring *A* (C1—C6) and the benzene ring D (C14–C19), respectively. Similarly, there exist π–π interactions with a distance of 3.7156 (14) Å between the centeroids of *Cg*5—*Cg*6^ii^ and *Cg*6—*Cg*5^ii^ [ii = 2 - *x*, -*y*, 1 - *z*], where *Cg*5 and *Cg*6 are the centroids of the benzene ring *E* (C20—C25) and the benzene ring H (C33–C38), respectively.

### S2. Experimental

For the preparation of title compound, 4-acetyl-3-methyl-1-phenyl-5-hydroxy pyrazole (0.218 g, 1 mmoL), 4-chlorobenzaldehyde (0.211 g, 1.5 mmoL) in glacial acetic acid (10 ml) and concentrated sulfuric acid (0.2 ml) were stirred at 353–360 K for 5 h. The reaction mixture was diluted with distilled water (50 ml). The precipitate was filtered, washed with methanol and dried. The crude product was purified by column chromatography using n-hexane and ethyl acetate mixtures as eluents. The product was recrystallized using n-hexane to afford red plates (yield 53%; m.p. 483 K).

### S3. Refinement

The H-atoms were positioned geometrically (C–H = 0.93–0.96 Å, O—H= 0.82 Å) and refined as riding with *U*_iso_(H) = *xU*_eq_(C, O), where *x* = 1.5 for methyl and hydroxy and *x* =1.2 for other H-atoms.

### Crystal data

**Table:**

C_19_H_15_ClN_2_O_2_	*Z* = 4
*M**_r_* = 338.78	*F*(000) = 704
Triclinic, *P*1	*D*_x_ = 1.392 Mg m^−^^3^
*a* = 11.3207 (6) Å	Mo *K*α radiation, λ = 0.71073 Å
*b* = 11.4044 (6) Å	Cell parameters from 4689 reflections
*c* = 15.2839 (9) Å	θ = 1.4–27.0°
α = 70.567 (3)°	µ = 0.25 mm^−^^1^
β = 70.925 (3)°	*T* = 296 K
γ = 62.621 (2)°	Plate, red
*V* = 1616.40 (16) Å^3^	0.35 × 0.28 × 0.16 mm

### Data collection

**Table:**

Bruker Kappa APEXII CCD diffractometer	6925 independent reflections
Radiation source: fine-focus sealed tube	4587 reflections with *I* > 2σ(*I*)
Graphite monochromator	*R*_int_ = 0.038
Detector resolution: 7.80 pixels mm^-1^	θ_max_ = 27.0°, θ_min_ = 1.4°
ω scans	*h* = −14→14
Absorption correction: multi-scan (*SADABS*; Bruker, 2005)	*k* = −14→14
*T*_min_ = 0.919, *T*_max_ = 0.963	*l* = −18→19
24363 measured reflections	

### Refinement

**Table:**

Refinement on *F*^2^	Primary atom site location: structure-invariant direct methods
Least-squares matrix: full	Secondary atom site location: difference Fourier map
*R*[*F*^2^ > 2σ(*F*^2^)] = 0.050	Hydrogen site location: inferred from neighbouring sites
*wR*(*F*^2^) = 0.155	H-atom parameters constrained
*S* = 1.02	*w* = 1/[σ^2^(*F*_o_^2^) + (0.0798*P*)^2^ + 0.3075*P*] where *P* = (*F*_o_^2^ + 2*F*_c_^2^)/3
6925 reflections	(Δ/σ)_max_ < 0.001
437 parameters	Δρ_max_ = 0.36 e Å^−^^3^
0 restraints	Δρ_min_ = −0.32 e Å^−^^3^

### Special details

**Table:**

Geometry. All e.s.d.'s (except the e.s.d. in the dihedral angle between two l.s. planes) are estimated using the full covariance matrix. The cell e.s.d.'s are taken into account individually in the estimation of e.s.d.'s in distances, angles and torsion angles; correlations between e.s.d.'s in cell parameters are only used when they are defined by crystal symmetry. An approximate (isotropic) treatment of cell e.s.d.'s is used for estimating e.s.d.'s involving l.s. planes.
Refinement. Refinement of *F*^2^ against ALL reflections. The weighted *R*-factor *wR* and goodness of fit *S* are based on *F*^2^, conventional *R*-factors *R* are based on *F*, with *F* set to zero for negative *F*^2^. The threshold expression of *F*^2^ > σ(*F*^2^) is used only for calculating *R*-factors(gt) *etc*. and is not relevant to the choice of reflections for refinement. *R*-factors based on *F*^2^ are statistically about twice as large as those based on *F*, and *R*-factors based on ALL data will be even larger.

### Fractional atomic coordinates and isotropic or equivalent isotropic displacement parameters (Å^2^)

**Table:**

	*x*	*y*	*z*	*U*_iso_*/*U*_eq_	
Cl1	−0.47686 (7)	−0.29735 (7)	0.36338 (6)	0.0759 (2)	
O1	0.30105 (14)	0.03074 (15)	0.37120 (13)	0.0633 (4)	
O2	0.17939 (15)	−0.11029 (15)	0.37314 (13)	0.0627 (4)	
H2A	0.2397	−0.0885	0.3718	0.094*	
N1	0.15162 (16)	0.25570 (17)	0.37444 (12)	0.0473 (4)	
N2	0.01106 (16)	0.33184 (17)	0.38151 (13)	0.0523 (5)	
C1	0.2367 (2)	0.3144 (2)	0.37686 (14)	0.0438 (5)	
C2	0.3765 (2)	0.2576 (2)	0.34316 (15)	0.0531 (5)	
H2	0.4150	0.1827	0.3160	0.064*	
C3	0.4570 (2)	0.3133 (3)	0.35038 (17)	0.0601 (6)	
H3	0.5506	0.2752	0.3283	0.072*	
C4	0.4017 (3)	0.4242 (3)	0.38966 (18)	0.0662 (7)	
H4	0.4573	0.4601	0.3951	0.079*	
C5	0.2636 (3)	0.4815 (2)	0.42084 (17)	0.0640 (6)	
H5	0.2255	0.5579	0.4461	0.077*	
C6	0.1805 (2)	0.4273 (2)	0.41528 (15)	0.0532 (5)	
H6	0.0870	0.4664	0.4373	0.064*	
C7	0.1839 (2)	0.1261 (2)	0.37349 (15)	0.0476 (5)	
C8	0.06103 (19)	0.1169 (2)	0.37778 (14)	0.0446 (5)	
C9	−0.0411 (2)	0.2509 (2)	0.38279 (15)	0.0476 (5)	
C10	−0.1907 (2)	0.3019 (2)	0.38925 (18)	0.0619 (6)	
H10A	−0.2325	0.3964	0.3902	0.093*	
H10B	−0.2297	0.2527	0.4464	0.093*	
H10C	−0.2060	0.2894	0.3354	0.093*	
C11	0.0633 (2)	−0.0054 (2)	0.37681 (15)	0.0471 (5)	
C12	−0.0535 (2)	−0.0281 (2)	0.37934 (15)	0.0486 (5)	
H12	−0.1397	0.0394	0.3917	0.058*	
C13	−0.0426 (2)	−0.1408 (2)	0.36480 (14)	0.0470 (5)	
H13	0.0452	−0.2058	0.3527	0.056*	
C14	−0.15161 (19)	−0.17573 (19)	0.36545 (13)	0.0411 (4)	
C15	−0.2886 (2)	−0.1023 (2)	0.40044 (15)	0.0484 (5)	
H15	−0.3129	−0.0279	0.4249	0.058*	
C16	−0.3879 (2)	−0.1390 (2)	0.39903 (15)	0.0504 (5)	
H16	−0.4789	−0.0891	0.4219	0.061*	
C17	−0.3518 (2)	−0.2496 (2)	0.36372 (15)	0.0488 (5)	
C18	−0.2180 (2)	−0.3242 (2)	0.32883 (15)	0.0496 (5)	
H18	−0.1947	−0.3984	0.3044	0.059*	
C19	−0.1191 (2)	−0.2870 (2)	0.33063 (14)	0.0469 (5)	
H19	−0.0284	−0.3377	0.3079	0.056*	
Cl2	1.30602 (7)	0.98559 (7)	0.13178 (6)	0.0820 (2)	
N3	0.75509 (18)	0.35396 (17)	0.12243 (13)	0.0507 (4)	
N4	0.68090 (19)	0.49510 (17)	0.10654 (14)	0.0556 (5)	
O3	0.97609 (16)	0.20330 (14)	0.14297 (13)	0.0664 (5)	
O4	1.11542 (15)	0.32575 (15)	0.14249 (13)	0.0629 (4)	
H4A	1.0921	0.2649	0.1481	0.094*	
C20	0.6945 (2)	0.2690 (2)	0.12269 (14)	0.0480 (5)	
C21	0.5784 (2)	0.3249 (3)	0.08691 (16)	0.0592 (6)	
H21	0.5397	0.4176	0.0630	0.071*	
C22	0.5202 (3)	0.2425 (3)	0.08689 (19)	0.0708 (7)	
H22	0.4415	0.2803	0.0632	0.085*	
C23	0.5763 (3)	0.1058 (3)	0.12117 (19)	0.0739 (7)	
H23	0.5369	0.0507	0.1200	0.089*	
C24	0.6914 (3)	0.0508 (3)	0.15718 (17)	0.0662 (7)	
H24	0.7294	−0.0420	0.1808	0.079*	
C25	0.7512 (2)	0.1304 (2)	0.15898 (16)	0.0578 (6)	
H25	0.8288	0.0922	0.1841	0.069*	
C26	0.8828 (2)	0.3216 (2)	0.13010 (15)	0.0499 (5)	
C27	0.8924 (2)	0.4451 (2)	0.12111 (15)	0.0474 (5)	
C28	0.7614 (2)	0.5477 (2)	0.10651 (15)	0.0498 (5)	
C29	0.7111 (3)	0.6984 (2)	0.09116 (18)	0.0636 (6)	
H29A	0.6187	0.7397	0.0833	0.095*	
H29B	0.7669	0.7314	0.0353	0.095*	
H29C	0.7154	0.7206	0.1451	0.095*	
C30	1.0133 (2)	0.4430 (2)	0.12793 (15)	0.0498 (5)	
C31	1.0370 (2)	0.5599 (2)	0.12068 (15)	0.0508 (5)	
H31	0.9718	0.6453	0.1022	0.061*	
C32	1.1478 (2)	0.5509 (2)	0.13923 (14)	0.0492 (5)	
H32	1.2114	0.4637	0.1558	0.059*	
C33	1.1824 (2)	0.6600 (2)	0.13693 (14)	0.0444 (5)	
C34	1.2924 (2)	0.6291 (2)	0.17330 (15)	0.0513 (5)	
H34	1.3428	0.5393	0.1982	0.062*	
C35	1.3295 (2)	0.7284 (2)	0.17355 (16)	0.0577 (6)	
H35	1.4032	0.7061	0.1990	0.069*	
C36	1.2560 (2)	0.8606 (2)	0.13566 (16)	0.0538 (5)	
C37	1.1451 (2)	0.8952 (2)	0.09963 (17)	0.0586 (6)	
H37	1.0956	0.9853	0.0747	0.070*	
C38	1.1081 (2)	0.7963 (2)	0.10069 (16)	0.0542 (6)	
H38	1.0323	0.8199	0.0770	0.065*	

### Atomic displacement parameters (Å^2^)

**Table:**

	*U*^11^	*U*^22^	*U*^33^	*U*^12^	*U*^13^	*U*^23^
Cl1	0.0599 (4)	0.0658 (4)	0.1194 (6)	−0.0307 (3)	−0.0291 (4)	−0.0219 (4)
O1	0.0380 (8)	0.0445 (8)	0.1090 (13)	−0.0038 (7)	−0.0233 (8)	−0.0291 (8)
O2	0.0411 (8)	0.0459 (9)	0.1043 (13)	−0.0087 (7)	−0.0231 (8)	−0.0249 (8)
N1	0.0358 (9)	0.0392 (9)	0.0648 (11)	−0.0090 (7)	−0.0129 (8)	−0.0149 (8)
N2	0.0338 (9)	0.0410 (9)	0.0739 (12)	−0.0062 (8)	−0.0120 (8)	−0.0142 (9)
C1	0.0432 (11)	0.0398 (10)	0.0479 (11)	−0.0167 (9)	−0.0102 (9)	−0.0079 (9)
C2	0.0468 (12)	0.0518 (13)	0.0585 (13)	−0.0193 (10)	−0.0050 (10)	−0.0156 (10)
C3	0.0516 (13)	0.0650 (15)	0.0670 (15)	−0.0308 (12)	−0.0108 (11)	−0.0085 (12)
C4	0.0781 (18)	0.0654 (16)	0.0728 (16)	−0.0422 (14)	−0.0256 (14)	−0.0061 (13)
C5	0.0792 (18)	0.0535 (14)	0.0684 (16)	−0.0278 (13)	−0.0195 (13)	−0.0177 (12)
C6	0.0535 (13)	0.0481 (12)	0.0564 (13)	−0.0178 (10)	−0.0073 (10)	−0.0165 (10)
C7	0.0412 (11)	0.0403 (11)	0.0605 (13)	−0.0111 (9)	−0.0139 (9)	−0.0139 (9)
C8	0.0364 (10)	0.0408 (11)	0.0571 (12)	−0.0112 (9)	−0.0157 (9)	−0.0113 (9)
C9	0.0365 (11)	0.0428 (11)	0.0591 (13)	−0.0113 (9)	−0.0128 (9)	−0.0095 (10)
C10	0.0408 (12)	0.0479 (13)	0.0874 (17)	−0.0077 (10)	−0.0162 (11)	−0.0148 (12)
C11	0.0385 (11)	0.0447 (12)	0.0584 (13)	−0.0120 (9)	−0.0152 (9)	−0.0128 (9)
C12	0.0395 (11)	0.0433 (11)	0.0620 (13)	−0.0115 (9)	−0.0151 (9)	−0.0128 (10)
C13	0.0388 (11)	0.0445 (12)	0.0552 (12)	−0.0129 (9)	−0.0122 (9)	−0.0104 (9)
C14	0.0386 (10)	0.0356 (10)	0.0467 (11)	−0.0120 (8)	−0.0124 (8)	−0.0064 (8)
C15	0.0429 (11)	0.0402 (11)	0.0624 (13)	−0.0112 (9)	−0.0114 (9)	−0.0187 (10)
C16	0.0354 (11)	0.0449 (12)	0.0672 (14)	−0.0089 (9)	−0.0120 (10)	−0.0165 (10)
C17	0.0455 (12)	0.0427 (11)	0.0610 (13)	−0.0184 (10)	−0.0199 (10)	−0.0047 (10)
C18	0.0557 (13)	0.0337 (10)	0.0603 (13)	−0.0161 (10)	−0.0151 (10)	−0.0106 (9)
C19	0.0408 (11)	0.0385 (11)	0.0560 (12)	−0.0111 (9)	−0.0085 (9)	−0.0118 (9)
Cl2	0.0742 (5)	0.0591 (4)	0.1263 (6)	−0.0358 (3)	−0.0237 (4)	−0.0197 (4)
N3	0.0475 (10)	0.0362 (9)	0.0649 (11)	−0.0116 (8)	−0.0182 (8)	−0.0075 (8)
N4	0.0510 (11)	0.0364 (9)	0.0718 (12)	−0.0076 (8)	−0.0201 (9)	−0.0095 (8)
O3	0.0548 (10)	0.0347 (8)	0.1091 (13)	−0.0077 (7)	−0.0308 (9)	−0.0159 (8)
O4	0.0520 (9)	0.0385 (8)	0.0979 (12)	−0.0100 (7)	−0.0209 (8)	−0.0205 (8)
C20	0.0513 (12)	0.0464 (12)	0.0463 (12)	−0.0206 (10)	−0.0108 (9)	−0.0071 (9)
C21	0.0584 (14)	0.0560 (14)	0.0602 (14)	−0.0151 (11)	−0.0224 (11)	−0.0100 (11)
C22	0.0643 (16)	0.083 (2)	0.0764 (17)	−0.0264 (14)	−0.0289 (13)	−0.0207 (14)
C23	0.0816 (19)	0.086 (2)	0.0763 (18)	−0.0487 (17)	−0.0184 (14)	−0.0195 (15)
C24	0.0820 (18)	0.0561 (14)	0.0696 (16)	−0.0380 (13)	−0.0227 (13)	−0.0021 (12)
C25	0.0619 (14)	0.0497 (13)	0.0643 (14)	−0.0257 (11)	−0.0241 (11)	0.0009 (11)
C26	0.0482 (12)	0.0410 (11)	0.0573 (13)	−0.0136 (10)	−0.0142 (10)	−0.0098 (9)
C27	0.0495 (12)	0.0368 (10)	0.0553 (12)	−0.0134 (9)	−0.0142 (9)	−0.0112 (9)
C28	0.0518 (13)	0.0371 (11)	0.0536 (13)	−0.0106 (10)	−0.0136 (10)	−0.0091 (9)
C29	0.0634 (15)	0.0384 (12)	0.0806 (17)	−0.0073 (11)	−0.0248 (13)	−0.0121 (11)
C30	0.0516 (13)	0.0395 (11)	0.0543 (13)	−0.0130 (10)	−0.0097 (10)	−0.0142 (9)
C31	0.0504 (13)	0.0397 (11)	0.0607 (13)	−0.0141 (10)	−0.0116 (10)	−0.0144 (10)
C32	0.0494 (12)	0.0395 (11)	0.0533 (12)	−0.0132 (9)	−0.0073 (10)	−0.0134 (9)
C33	0.0409 (11)	0.0390 (10)	0.0490 (12)	−0.0132 (9)	−0.0055 (9)	−0.0121 (9)
C34	0.0472 (12)	0.0406 (11)	0.0593 (13)	−0.0120 (9)	−0.0149 (10)	−0.0066 (10)
C35	0.0464 (13)	0.0573 (14)	0.0711 (15)	−0.0184 (11)	−0.0197 (11)	−0.0116 (11)
C36	0.0488 (13)	0.0458 (12)	0.0663 (14)	−0.0198 (10)	−0.0075 (10)	−0.0141 (10)
C37	0.0526 (13)	0.0380 (11)	0.0812 (16)	−0.0133 (10)	−0.0209 (12)	−0.0079 (11)
C38	0.0461 (12)	0.0442 (12)	0.0727 (15)	−0.0135 (10)	−0.0219 (11)	−0.0101 (11)

### Geometric parameters (Å, º)

**Table:**

Cl1—C17	1.736 (2)	Cl2—C36	1.738 (2)
O1—C7	1.269 (2)	N3—C26	1.354 (3)
O2—C11	1.309 (2)	N3—N4	1.409 (2)
O2—H2A	0.8200	N3—C20	1.419 (3)
N1—C7	1.353 (3)	N4—C28	1.301 (3)
N1—N2	1.406 (2)	O3—C26	1.274 (2)
N1—C1	1.418 (2)	O4—C30	1.312 (2)
N2—C9	1.298 (3)	O4—H4A	0.8200
C1—C6	1.383 (3)	C20—C21	1.379 (3)
C1—C2	1.391 (3)	C20—C25	1.391 (3)
C2—C3	1.376 (3)	C21—C22	1.373 (3)
C2—H2	0.9300	C21—H21	0.9300
C3—C4	1.372 (4)	C22—C23	1.369 (4)
C3—H3	0.9300	C22—H22	0.9300
C4—C5	1.371 (3)	C23—C24	1.371 (4)
C4—H4	0.9300	C23—H23	0.9300
C5—C6	1.377 (3)	C24—C25	1.370 (3)
C5—H5	0.9300	C24—H24	0.9300
C6—H6	0.9300	C25—H25	0.9300
C7—C8	1.422 (3)	C26—C27	1.419 (3)
C8—C11	1.388 (3)	C27—C30	1.395 (3)
C8—C9	1.439 (3)	C27—C28	1.436 (3)
C9—C10	1.496 (3)	C28—C29	1.501 (3)
C10—H10A	0.9600	C29—H29A	0.9600
C10—H10B	0.9600	C29—H29B	0.9600
C10—H10C	0.9600	C29—H29C	0.9600
C11—C12	1.447 (3)	C30—C31	1.440 (3)
C12—C13	1.321 (3)	C31—C32	1.326 (3)
C12—H12	0.9300	C31—H31	0.9300
C13—C14	1.457 (3)	C32—C33	1.454 (3)
C13—H13	0.9300	C32—H32	0.9300
C14—C19	1.386 (3)	C33—C34	1.379 (3)
C14—C15	1.398 (3)	C33—C38	1.400 (3)
C15—C16	1.376 (3)	C34—C35	1.378 (3)
C15—H15	0.9300	C34—H34	0.9300
C16—C17	1.373 (3)	C35—C36	1.371 (3)
C16—H16	0.9300	C35—H35	0.9300
C17—C18	1.375 (3)	C36—C37	1.373 (3)
C18—C19	1.378 (3)	C37—C38	1.366 (3)
C18—H18	0.9300	C37—H37	0.9300
C19—H19	0.9300	C38—H38	0.9300
			
C11—O2—H2A	109.5	C26—N3—N4	110.82 (17)
C7—N1—N2	110.82 (16)	C26—N3—C20	129.58 (18)
C7—N1—C1	128.74 (17)	N4—N3—C20	119.48 (17)
N2—N1—C1	120.22 (16)	C28—N4—N3	106.55 (17)
C9—N2—N1	106.60 (16)	C30—O4—H4A	109.5
C6—C1—C2	119.7 (2)	C21—C20—C25	120.0 (2)
C6—C1—N1	119.60 (18)	C21—C20—N3	119.53 (19)
C2—C1—N1	120.64 (19)	C25—C20—N3	120.5 (2)
C3—C2—C1	119.3 (2)	C22—C21—C20	119.4 (2)
C3—C2—H2	120.4	C22—C21—H21	120.3
C1—C2—H2	120.4	C20—C21—H21	120.3
C4—C3—C2	121.1 (2)	C23—C22—C21	121.0 (2)
C4—C3—H3	119.4	C23—C22—H22	119.5
C2—C3—H3	119.4	C21—C22—H22	119.5
C5—C4—C3	119.3 (2)	C22—C23—C24	119.3 (3)
C5—C4—H4	120.3	C22—C23—H23	120.4
C3—C4—H4	120.3	C24—C23—H23	120.4
C4—C5—C6	120.9 (2)	C25—C24—C23	121.1 (2)
C4—C5—H5	119.6	C25—C24—H24	119.4
C6—C5—H5	119.6	C23—C24—H24	119.4
C5—C6—C1	119.7 (2)	C24—C25—C20	119.1 (2)
C5—C6—H6	120.2	C24—C25—H25	120.4
C1—C6—H6	120.2	C20—C25—H25	120.4
O1—C7—N1	126.47 (19)	O3—C26—N3	126.6 (2)
O1—C7—C8	126.7 (2)	O3—C26—C27	126.8 (2)
N1—C7—C8	106.78 (17)	N3—C26—C27	106.63 (18)
C11—C8—C7	119.71 (18)	C30—C27—C26	119.56 (19)
C11—C8—C9	136.05 (19)	C30—C27—C28	135.7 (2)
C7—C8—C9	104.24 (18)	C26—C27—C28	104.70 (19)
N2—C9—C8	111.55 (18)	N4—C28—C27	111.28 (19)
N2—C9—C10	119.92 (18)	N4—C28—C29	119.4 (2)
C8—C9—C10	128.53 (19)	C27—C28—C29	129.3 (2)
C9—C10—H10A	109.5	C28—C29—H29A	109.5
C9—C10—H10B	109.5	C28—C29—H29B	109.5
H10A—C10—H10B	109.5	H29A—C29—H29B	109.5
C9—C10—H10C	109.5	C28—C29—H29C	109.5
H10A—C10—H10C	109.5	H29A—C29—H29C	109.5
H10B—C10—H10C	109.5	H29B—C29—H29C	109.5
O2—C11—C8	118.66 (18)	O4—C30—C27	118.5 (2)
O2—C11—C12	115.98 (19)	O4—C30—C31	116.0 (2)
C8—C11—C12	125.37 (19)	C27—C30—C31	125.50 (19)
C13—C12—C11	122.6 (2)	C32—C31—C30	122.8 (2)
C13—C12—H12	118.7	C32—C31—H31	118.6
C11—C12—H12	118.7	C30—C31—H31	118.6
C12—C13—C14	127.80 (19)	C31—C32—C33	128.2 (2)
C12—C13—H13	116.1	C31—C32—H32	115.9
C14—C13—H13	116.1	C33—C32—H32	115.9
C19—C14—C15	118.03 (19)	C34—C33—C38	117.9 (2)
C19—C14—C13	119.07 (18)	C34—C33—C32	118.95 (19)
C15—C14—C13	122.90 (19)	C38—C33—C32	123.16 (19)
C16—C15—C14	120.7 (2)	C35—C34—C33	121.5 (2)
C16—C15—H15	119.7	C35—C34—H34	119.3
C14—C15—H15	119.7	C33—C34—H34	119.3
C17—C16—C15	119.63 (19)	C36—C35—C34	119.0 (2)
C17—C16—H16	120.2	C36—C35—H35	120.5
C15—C16—H16	120.2	C34—C35—H35	120.5
C16—C17—C18	121.2 (2)	C35—C36—C37	121.1 (2)
C16—C17—Cl1	119.67 (17)	C35—C36—Cl2	119.22 (19)
C18—C17—Cl1	119.13 (17)	C37—C36—Cl2	119.69 (18)
C17—C18—C19	118.8 (2)	C38—C37—C36	119.5 (2)
C17—C18—H18	120.6	C38—C37—H37	120.2
C19—C18—H18	120.6	C36—C37—H37	120.2
C18—C19—C14	121.62 (19)	C37—C38—C33	121.0 (2)
C18—C19—H19	119.2	C37—C38—H38	119.5
C14—C19—H19	119.2	C33—C38—H38	119.5
			
C7—N1—N2—C9	−1.3 (2)	C26—N3—N4—C28	−1.3 (2)
C1—N1—N2—C9	−176.40 (17)	C20—N3—N4—C28	−177.68 (18)
C7—N1—C1—C6	−153.7 (2)	C26—N3—C20—C21	−161.1 (2)
N2—N1—C1—C6	20.4 (3)	N4—N3—C20—C21	14.5 (3)
C7—N1—C1—C2	24.7 (3)	C26—N3—C20—C25	19.1 (3)
N2—N1—C1—C2	−161.22 (19)	N4—N3—C20—C25	−165.3 (2)
C6—C1—C2—C3	1.3 (3)	C25—C20—C21—C22	−0.5 (3)
N1—C1—C2—C3	−177.10 (19)	N3—C20—C21—C22	179.8 (2)
C1—C2—C3—C4	−0.4 (3)	C20—C21—C22—C23	−0.5 (4)
C2—C3—C4—C5	−1.0 (4)	C21—C22—C23—C24	0.9 (4)
C3—C4—C5—C6	1.6 (4)	C22—C23—C24—C25	−0.3 (4)
C4—C5—C6—C1	−0.7 (3)	C23—C24—C25—C20	−0.6 (4)
C2—C1—C6—C5	−0.8 (3)	C21—C20—C25—C24	1.0 (3)
N1—C1—C6—C5	177.64 (19)	N3—C20—C25—C24	−179.2 (2)
N2—N1—C7—O1	−177.3 (2)	N4—N3—C26—O3	−178.7 (2)
C1—N1—C7—O1	−2.7 (4)	C20—N3—C26—O3	−2.8 (4)
N2—N1—C7—C8	1.4 (2)	N4—N3—C26—C27	1.3 (2)
C1—N1—C7—C8	175.97 (19)	C20—N3—C26—C27	177.25 (19)
O1—C7—C8—C11	−2.0 (4)	O3—C26—C27—C30	−1.1 (4)
N1—C7—C8—C11	179.28 (19)	N3—C26—C27—C30	178.85 (19)
O1—C7—C8—C9	177.7 (2)	O3—C26—C27—C28	179.2 (2)
N1—C7—C8—C9	−1.0 (2)	N3—C26—C27—C28	−0.9 (2)
N1—N2—C9—C8	0.7 (2)	N3—N4—C28—C27	0.7 (2)
N1—N2—C9—C10	−179.56 (19)	N3—N4—C28—C29	−179.97 (19)
C11—C8—C9—N2	179.9 (2)	C30—C27—C28—N4	−179.5 (2)
C7—C8—C9—N2	0.2 (2)	C26—C27—C28—N4	0.1 (3)
C11—C8—C9—C10	0.1 (4)	C30—C27—C28—C29	1.2 (4)
C7—C8—C9—C10	−179.6 (2)	C26—C27—C28—C29	−179.2 (2)
C7—C8—C11—O2	0.8 (3)	C26—C27—C30—O4	0.1 (3)
C9—C8—C11—O2	−178.8 (2)	C28—C27—C30—O4	179.7 (2)
C7—C8—C11—C12	−179.00 (19)	C26—C27—C30—C31	−179.7 (2)
C9—C8—C11—C12	1.3 (4)	C28—C27—C30—C31	−0.1 (4)
O2—C11—C12—C13	−9.7 (3)	O4—C30—C31—C32	−8.3 (3)
C8—C11—C12—C13	170.2 (2)	C27—C30—C31—C32	171.5 (2)
C11—C12—C13—C14	180.00 (19)	C30—C31—C32—C33	−178.36 (19)
C12—C13—C14—C19	167.5 (2)	C31—C32—C33—C34	169.7 (2)
C12—C13—C14—C15	−13.0 (3)	C31—C32—C33—C38	−9.3 (3)
C19—C14—C15—C16	−0.8 (3)	C38—C33—C34—C35	−0.5 (3)
C13—C14—C15—C16	179.66 (19)	C32—C33—C34—C35	−179.59 (19)
C14—C15—C16—C17	0.6 (3)	C33—C34—C35—C36	−0.8 (3)
C15—C16—C17—C18	−0.6 (3)	C34—C35—C36—C37	1.4 (4)
C15—C16—C17—Cl1	179.14 (16)	C34—C35—C36—Cl2	−177.63 (17)
C16—C17—C18—C19	0.7 (3)	C35—C36—C37—C38	−0.6 (4)
Cl1—C17—C18—C19	−179.03 (15)	Cl2—C36—C37—C38	178.40 (18)
C17—C18—C19—C14	−0.9 (3)	C36—C37—C38—C33	−0.8 (4)
C15—C14—C19—C18	0.9 (3)	C34—C33—C38—C37	1.3 (3)
C13—C14—C19—C18	−179.52 (18)	C32—C33—C38—C37	−179.7 (2)

### Hydrogen-bond geometry (Å, º)

**Table:**

*D*—H···*A*	*D*—H	H···*A*	*D*···*A*	*D*—H···*A*
O2—H2*A*···O1	0.82	1.78	2.542 (2)	153
C16—H16···O1^i^	0.93	2.47	3.239 (2)	140
O4—H4*A*···O3	0.82	1.78	2.540 (2)	153
C37—H37···O3^ii^	0.93	2.61	3.314 (3)	133

Symmetry codes: (i) *x*−1, *y*, *z*; (ii) *x*, *y*+1, *z*.
