# Supplementary figures and images for: Crystal structure of (4Z)-4-[(2E)-3-(4-chloro­phen­yl)-1-hy­droxy­prop-2-en-1-yl­idene]-5-methyl-2-phenyl-1H-pyrazol-5(4H)-one
Source: Acta Crystallogr E Crystallogr Commun. 2015 May 13;71(Pt 6):o393–4. doi: 10.1107/S205698901500883X (PMC4459345; doi:10.1107/S205698901500883X)

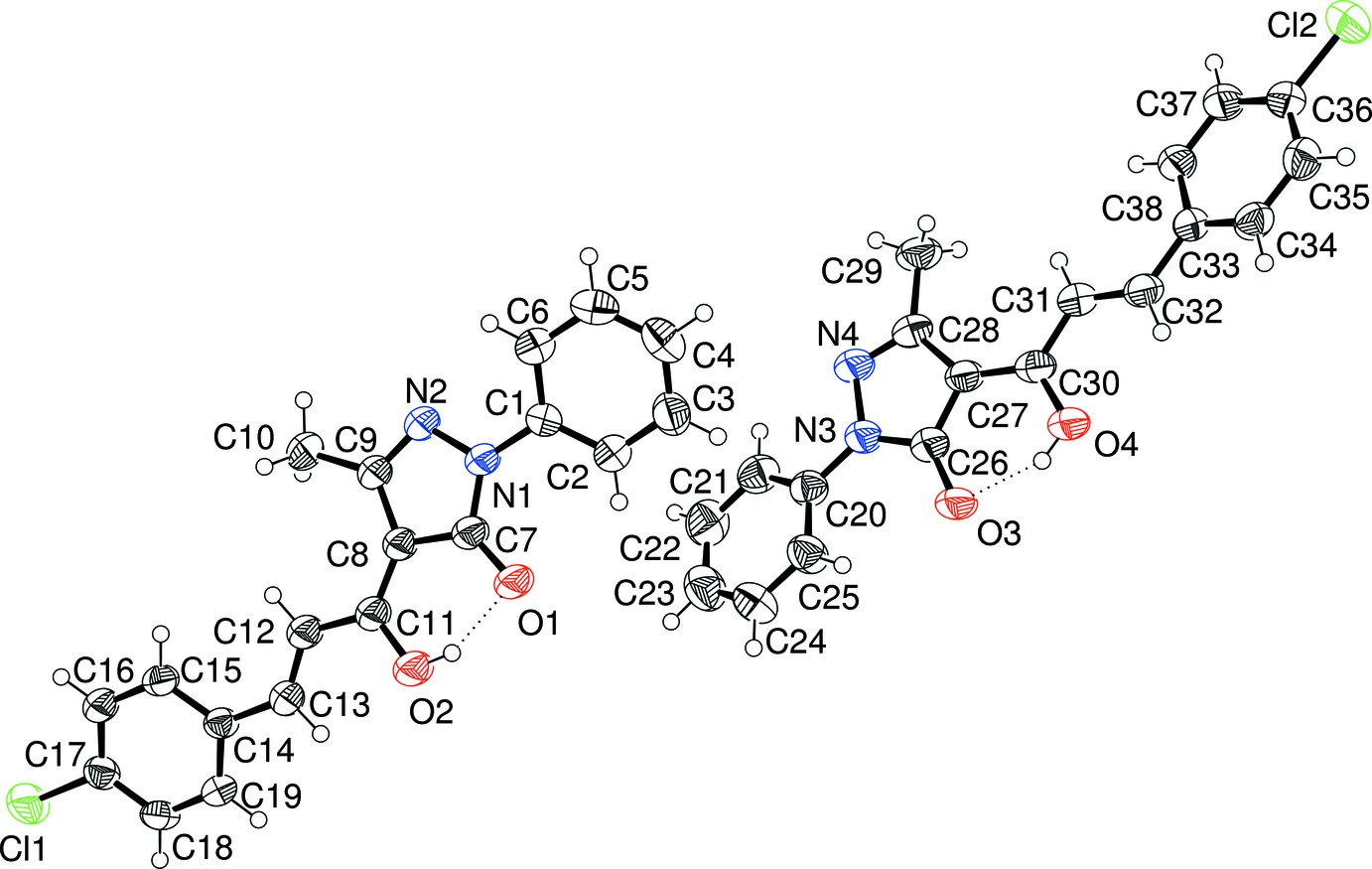

Supplement: Supplementary file 3 [file e-71-0o393-fig1.tif]

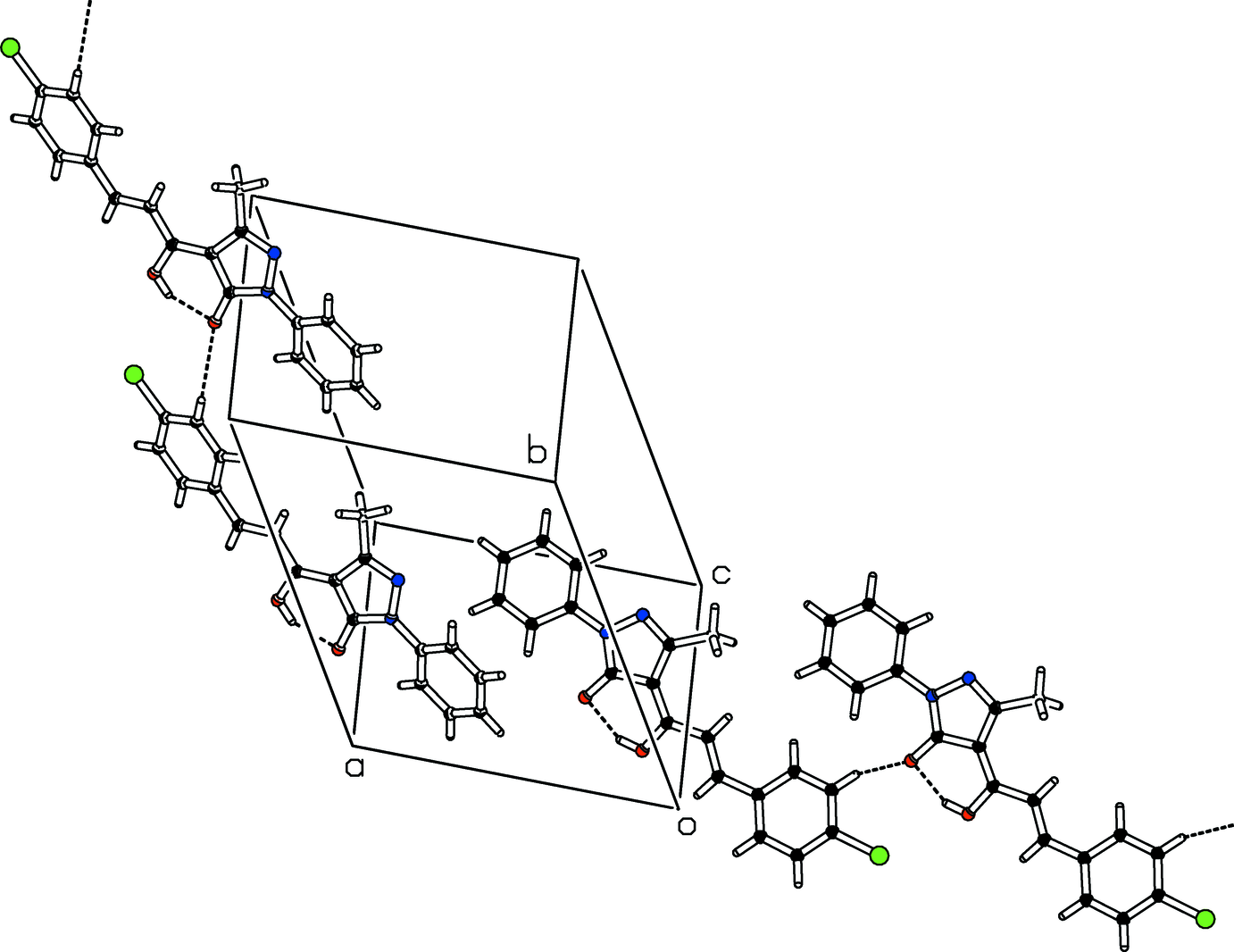

Supplement: Supplementary file 4 [file e-71-0o393-fig2.tif]
